# Microbial Adaptation to Enhance Stress Tolerance

**DOI:** 10.3389/fmicb.2022.888746

**Published:** 2022-04-27

**Authors:** Yong-Shui Tan, Ren-Kuan Zhang, Zhi-Hua Liu, Bing-Zhi Li, Ying-Jin Yuan

**Affiliations:** ^1^Frontiers Science Center for Synthetic Biology and Key Laboratory of Systems Bioengineering (Ministry of Education), School of Chemical Engineering and Technology, Tianjin University, Tianjin, China; ^2^Synthetic Biology Research Platform, Collaborative Innovation Center of Chemical Science and Engineering (Tianjin), Tianjin University, Tianjin, China

**Keywords:** adaptation, microorganisms, transcription factors, epigenetic modification, cross-protection against stress, synthetic biology

## Abstract

Microbial cell factories have been widely used in the production of various chemicals. Although synthetic biology is useful in improving the cell factories, adaptation is still widely applied to enhance its complex properties. Adaptation is an important strategy for enhancing stress tolerance in microbial cell factories. Adaptation involves gradual modifications of microorganisms in a stressful environment to enhance their tolerance. During adaptation, microorganisms use different mechanisms to enhance non-preferred substrate utilization and stress tolerance, thereby improving their ability to adapt for growth and survival. In this paper, the progress on the effects of adaptation on microbial substrate utilization capacity and environmental stress tolerance are reviewed, and the mechanisms involved in enhancing microbial adaptive capacity are discussed.

## Introduction

With the help of synthetic biology, microorganisms will produce around 30% of chemicals, biofuels and materials from renewable resources by 2030 (Lee et al., 2019; Yuan and Alper, 2019; Zeng, 2019; Zhang et al., 2021). However, microorganisms face complex and diverse substrates and harmful conditions during the production process, severely limiting the widespread use of microbial production in renewable energy (Huang et al., 2021). Although synthetic biology significantly improves strain development via the design-build-test-learn cycle, the efficiency of modifying and improving complex characteristics, such as tolerance to stress and speed of growth, is difficult to achieve due to the complex metabolic and regulatory networks involved. Therefore, adaptation is still widely used as a common approach to improve the performance of microbes (Sandberg et al., 2019).

**FIGURE 1:**
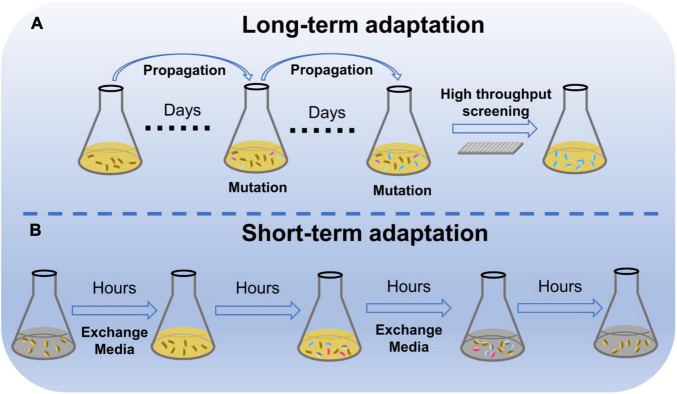
Microbial stress adaptation. **(A)** Schematic diagram of long-term adaptation of microorganisms to their environment. **(B)** Schematic diagram of short-term adaptation of microorganisms to their environment.

Adaptation is widely used to enhance performance of engineered strains due to its effectiveness and universality (Figure 1A). Microbial adaptation can improve the utilization of non-preferred substrates, while long-term adaptation of xylose-metabolizing yeast in a xylose culture enhanced the rate of xylose metabolism (Zha et al., 2014; Qi et al., 2015). The short-term adaptation of *Saccharomyces cerevisiae* in a galactose environment realized rapid utilization of galactose in subsequent generations (Stockwell et al., 2015). In addition, adaptation can rapidly improve tolerance to stress. adaption of *Saccharomyces eubayanus* in the culture with ethanol boosted the strain growth under ethanol condition (Mardones et al., 2021). Adaptation exhibits a great ability to improve microbial performance in a short time.

The mechanisms of microbial adaptation have attracted much attention recently. When microorganisms undergo long- and short-term adaptation, different mechanisms enhance the strain performance. Genomic mutations during adaptation could lead to fitness to the stressful environment by increasing the yield of the product or growth rate of the strain (Smith and Johnson, 2000; Sheppard et al., 2018). Sun et al. reported a novel stress-induced, error-prone Okazaki fragment that explains possible reasons for generating mutations, counteracting replication defects, and promoting cell evolution and survival (Sun et al., 2021). During short-term adaptation the fitness may be related to long-lasting protein residues, epigenetic modifications and cross-protection against different stresses (Kundu and Peterson, 2010; Goudarzi et al., 2016). This paper will provide an overview of recent advances in the applications and mechanisms of microbial adaptation.

## Adaptation to Improve Microbial Performance

Synthetic biology aims to redesign metabolic pathways and broaden the spectrum of products (e.g., protocatechuic acid, paclitaxel, opioids) (Guan et al., 2020; Tong et al., 2021; Zhang et al., 2021). However, the new substrates and products usually affect the performance of these artificial cell factories. Adaptation can enable microbial cell factories and enhance the conversion performance on different substrates (e.g., xylose, arabinose) (Li et al., 2019; Nijland and Driessen, 2020; Sun et al., 2020) and tolerance of stresses (Lian et al., 2018; Das et al., 2020; Guirimand et al., 2021).

### Adaptation to Enhance the Utilization of Non-preferred Substrates

With the development of synthetic biology, microbial cell factories can be designed to convert some non-preferred carbon sources from lignocelluloses, such as xylose and other pentoses derived from hydrolysis of lignocellulosic biomass (Li et al., 2020). Adaptation of microbial cell factories with the heterologous metabolic pathways for non-preferred carbon sources is usually able to improve the utilization efficiency of non-preferred carbon sources. *S. cerevisiae* is one of the most popular microbes for lignocellulosic ethanol production, but *S. cerevisiae* does not possess xylose metabolic pathways. Heterologous expression of the xylose reductase and xylitol dehydrogenase pathway enabled *S. cerevisiae* to utilize xylose (Zha et al., 2014). Xylose consumption increased slightly through regulation of several known key genes such as *RPE1*, *TKL1*, and *RKI1* in the pentose phosphate pathway (Zha et al., 2014; Qi et al., 2015). Adaptation of this xylose metabolizing *S. cerevisiae* strain in medium with 2% xylose as the sole carbon source for 12 consecutive passages resulted in rapid xylose utilization by the adapted strain and led to a 110% increase in isobutanol production (Promdonkoy et al., 2020). Adapted in xylose media for seven consecutive passages, xylose utilization of strain SyBE005 was significantly improved and ethanol production increased 2.6-fold (Zha et al., 2014). With adaptation for 60 days, xylose-utilizing yeast with modifications of xylose transporter gene and genes in phosphate pentose pathway improved strain growth more than threefolds in xylose media (Li et al., 2021). The efficiency of cellobiose utilization can also be improved through adaptations. After adaptation of 12 consecutive passages in a medium containing 80 g/L cellobiose, *S. cerevisiae* with the heterologous cellobiose metabolic pathway significantly increased the rate of cellobiose consumption and ethanol production (Oh Eun et al., 2016).

In addition to the enhanced utilization of non-preferred substrates by microorganisms after several days of passaging, microorganisms accelerated the utilization of non-preferred substrates after transient stimulation in a non-preferred substrate environment (Figure 1B). If *S. cerevisiae* is adapted for a short period of 12 h in a medium with galactose as the sole carbon source, the cells can take up the galactose and use it after enzymatic modification. Furthermore, the adapted cells can still rapidly utilize galactose after seven divisions (Stockwell et al., 2015). The viability of *S. cerevisiae* was improved through short-term adaptation in media lacking inositol, and its offspring still grew well in media lacking inositol (Light et al., 2013). Similarly, yeast undergoes rapid adaptation to non-preferred substrate during short-term adaptation between glucose and maltose, glucose and glycerol, and *Escherichia coli* between glucose and galactose (Novak et al., 2004; Ozbudak et al., 2004; Cerulus et al., 2018). Hence, short-term adaptation may also provide microorganisms with the ability to rapidly fit substrate changes, which can be passed on to the offspring.

Overall, adaptation compensates for the lack of knowledge of microbial on the utilization of non-preferred substrates. Microorganisms may activate and inhibit specific functions in the process of adapting to non-preferred substrates. Therefore, adaptation can rapidly improve microorganisms’ ability to utilize non-preferred substrates.

### Adaptation to Improve Stress Tolerance

During the fermentation process of yeast using lignocellulose to produce ethanol, microorganisms were affected by the stress of inhibitors such as furfural, phenol and acetic acid (FAP). Therefore, high-temperature environments and the potential presence of various toxic substances in the living environment can affect the survival of microorganisms as well as synthesis of chemicals and biofuels. As tolerance is a complex trait, it is not well-controlled by a single gene or few genes (Wang et al., 2015, 2020; Gulck et al., 2020; Qin et al., 2020). Thus, adaptation can enhance the tolerance of microorganisms to stressful environments on multiple scales, and is a common means for microorganisms to strengthen stress responses and overcome survival limitations.

The adaptation of *S. cerevisiae* for bioethanol production is promising. The *S. cerevisiae* was adapted at an initial concentration of 40% FAP mixed inhibitor (phenol 0.5 g/L, furfural 1.3 g/L, and acetic acid 5.3 g/L) before the inhibitor concentration was gradually increased to 100% over the course of the adaptation process for 65 days. The adapted strains were fermented at a concentration of 50% FAP mixed inhibitor. The ethanol yield of the adapted strains was 80% higher than the parental strains, and was able to eliminate furfural from the medium in a short period of time (Li et al., 2019). After adaptation with acetic acid and low pH conditions for 1 year, the growth rate of the adapted yeast increased to about 1.5 folds in 3 g/L of acetic acid condition compared with the parent strain (Salas-Navarrete et al., 2022). The adaptation of *Yarrowia lipolytica* strain led to tolerance of 1.5 g/L ferulic acid, whereas 0.5 g/L ferulic acid could cause about 90% lethality of the parental strain (Wang et al., 2021b). Furthermore, adaptation has been applied to microbes to overcome other stresses. The engineered yeast was adapted with oxidative stress to improve the tolerance and the production of β*-*caryophyllene (Godara and Kao, 2021). Adaptation of *E. coli* with the formic acid decreased the doubling time from 70 to 8 h (Kim et al., 2020). After 120 generations of adaptation of *Eubacterium limosum* under CO culture condition, the growth rate increased by 1.44-fold (Kang et al., 2020). These studies highlight that the toxic environment can inhibit microbial growth, while long-term adaptation of microorganisms can significantly improve their ability to ferment or metabolize toxic substances.

Short-term adaptation can also strengthen microorganisms’ ability to adapt to environmental stresses. Short-term adaptation of yeast was carried out for 8 min using a medium containing 1 M sorbitol. The yeast showed different behaviors in response to repeated stress 4 h apart, where a faster stress response rate was observed during the second time compared to the first time (Ben Meriem et al., 2019). Due to the lack of theoretical information on the genetic manipulation of wild-type microorganisms, it is difficult for wild-type microorganisms to resist the harsh environment through genetic engineering. Therefore, adaptation is a simple method that can effectively enhance the adaptability of microorganisms to the environment. With the constant development of low-cost, high-throughput DNA sequencing and bioinformatics, adapted strains can analyze the causes of strain changes using bioinformatics technology (Shendure et al., 2017; Šoštarić et al., 2021). Based on traditional genetic engineering techniques, adaptation can boost microorganisms’ performance to modify their adaptability to various environmental stresses, resulting in more efficient production strains.

## Mechanisms of Adaptation

Some of the performance improvements of strains caused by adaptation are long-term and stable, and some are short-term (Bagamery et al., 2020; Lee et al., 2020). The long-term stability is usually caused by gene mutation (Peter et al., 2018; Sheppard et al., 2018; Steensels et al., 2019), while the mechanism of short-term adaptation is more complex, and still under study. According to existing research, adaptation enhances short-term performance through several mechanisms. Good traits produced by short-term adaptation of microorganisms can be stably inherited for several generations and the microorganism gradually loses this phenotype as the number of generations increases. Based on the characteristics of short-term retention of traits, it is inferred that short-term adaptation may cause changes in tolerance through mechanisms such as residue of degradable proteins and epigenetic modifications.

### Adaptation Mechanisms Based on Long Retention Proteins

**FIGURE 2:**
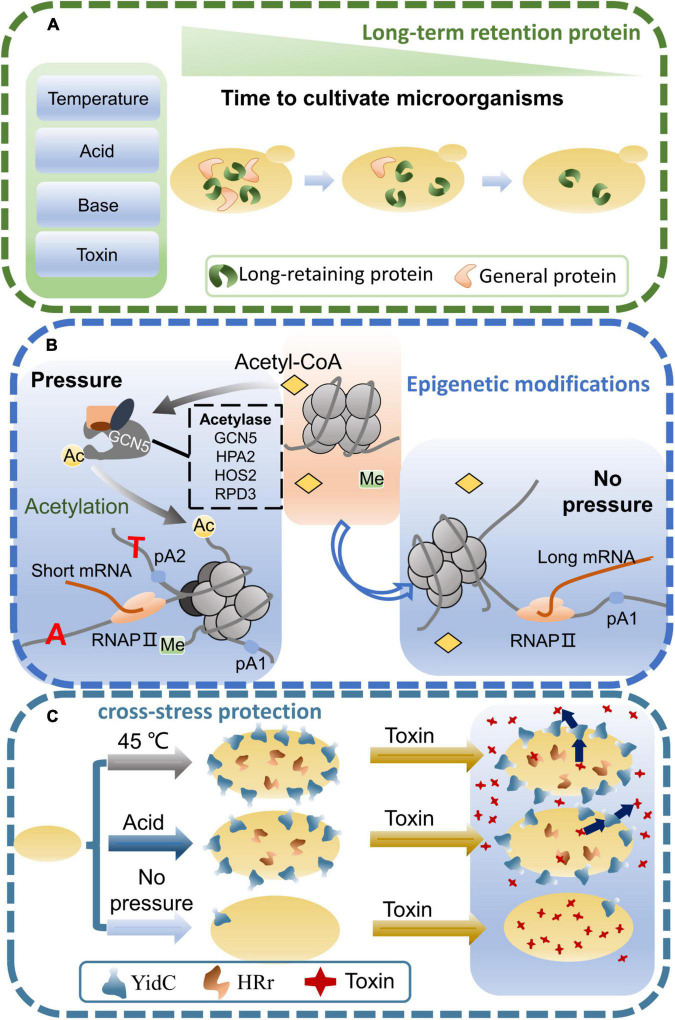
Mechanisms of microbial stress adaptation. **(A)** When microorganisms are subjected to environmental stress, the long-retaining proteins are produced to protect the cells for a long time. **(B)** Effects of environmental stress on microbial epigenetic modifications. When microorganisms are stimulated by external environmental stress, epigenetic modifications are altered, affecting transcription and translation processes. **(C)** Microorganisms are adapted to a stressful environment, resulting in cross protection against stress. Growth advantages can also be shown when switching to other stressful environments.

In the microorganisms, there may be long retention proteins that can enhance environmental tolerance (Figure 2A). Transcription factors may be an important long retention protein as they can regulate cells to maintain their physiological functions in a stress environment (Guan et al., 2012). Moreover, the interaction of transcription factors and DNA may also regulate complex metabolic networks. The retention time of transcription factors may determine the maintenance time of the new phenotypes. Therefore, the trait changes of short-term adapted strains may be closely related to the retention time of transcription factors. For instance, when *S. cerevisiae* was acclimated to galactose in the short term, the expression levels of galactose-metabolizing genes varied with glucose and galactose concentrations (Lohr et al., 1995). The GAL gene is slowly expressed when the yeast is first adapted in galactose for a short period of time. When galactose induction was repeated within a short time, yeast expressed GAL proteins at a faster rate and this response persisted for several generations in the absence of galactose. An in-depth analysis further revealed that previous adaptation mainly affected the expression of the transcription factors GAL3 and GAL1 (Kundu and Peterson, 2010). In addition, the nuclei of *S. cerevisiae* without galactose acclimation were put into the cytoplasm of the acclimated cells, which also resulted in enhanced galactose metabolism. This indicates that the cytoplasm may contain some substances that can regulate the metabolism of galactose after the cells are induced by galactose (Zacharioudakis et al., 2007).

During the short-term adaptation of microorganisms, transcription factors may also bind to specific promoters or enhancers, thereby inducing gene transcription to enhance stress tolerance (Weake and Workman, 2010). *S. cerevisiae* senses changes in energy and nutrition, thus regulating the expression of related genes through signal transduction pathways that may be influenced by transcription factors (González and Hall, 2017; González et al., 2020). *S. cerevisiae* was adapted in an environment lacking inositol, and the emergence of new traits was found to be related to the expression of the *INO1* gene (Wang et al., 2015). The expression of *INO1* gene is regulated by a variety of factors, including transcription factors SFL1, H2A.Z (Light et al., 2010; Brickner et al., 2015). When transcription factors and regulatory elements bind, it is likely to alter the structure of the chromosome and increase the accessibility of promoter regions on the chromosome (Weake and Workman, 2010). Therefore, transcription factors activated by environmental stress may bind to chromatin at specific binding sites. When cells are adapted for a short period of time, transcription factors bind to these loci, causing chromatin changes that are maintained for some time (Light et al., 2010). Hence, some long-retaining proteins remaining in microorganisms may play important regulatory roles during short-term adaptation.

### Mechanisms of Adaptation Based on Epigenetic Modifications

Epigenetic modifications can transiently regulate gene expression when microorganisms are stimulated by stressful environments (Figure 2B). However, only one epigenetically modified strand of DNA is contained in the daughter cell, and the offspring will no longer contain the modified DNA strand when the passage number increases (Radman-Livaja et al., 2011). Similarly, after short-term adaptation of microorganisms in a stressful environment, their tolerance traits gradually disappeared with an increasing passage number. Thus, there may be a close link between epigenetic modifications and short-term adaptation of microorganisms, and epigenetic modifications may play a key regulatory role in the adaptation process.

Microorganisms can respond to transient changes in their environment using heat shock responses, oxidative stress responses and DNA damage responses (Weinhouse, 2021). Epigenetic modification studies of these responses demonstrated that epigenetic modifications may lead to changes in phenotype (Henikoff and Greally, 2016; Harvey et al., 2018). Epigenetic modifications may affect DNA–histone interactions, nucleosome interactions and alter chromatin structure by modifying specific sites (Shogren-Knaak et al., 2006). However, nucleosomes are made up of histones H2A, H2B, H3, and H4, which are entwined with DNA strands to form higher order structures (Hauer and Gasser, 2017). For example, the acetylation of histones H3K14 and H3K9 facilitates the repair of UV-damaged DNA (Yu et al., 2005; Duan and Smerdon, 2014). Epigenetic modification provides a more transcriptionally efficient chromatin structure by converting chromatin into transcriptionally active euchromatin or inactive heterochromatin (Kouzarides, 2007). Therefore, the degree of sparseness of higher-order structures affects DNA transfer, limiting the ability of DNA to bind with transcription factors (Li et al., 2007; Marsano et al., 2019).

Microorganisms have various epigenetic modifications, such as lysine acetylation, serine phosphorylation, and lysine ubiquitination. Among them, lysine acetylation is involved in various cellular metabolisms and the transcription and translation of thousands of proteins (Goudarzi et al., 2016). Histones are epigenetically modified after previous short-term exposure of microorganisms to environmental stress, and the relevant genes are transcribed more rapidly when subjected to secondary stress (Brickner et al., 2007; Feil and Fraga, 2012; Rienzo et al., 2015). In transcriptional regulation, histone modifications can maintain the integrity of the genome and contribute to the stable inheritance of genetic information in daughter cells. In eukaryotes, epigenetic modifications have a direct effect on mitotic mutation rates, and two histone modifications (H3K9me3 and H3K27me3) associated with heterochromatin were shown to be associated with gene mutations. By correlating mutations with epigenetic modifications, it was found that H3K27me3 affects mutations by inducing replication stress. However, H3K27me3 modifications only affect local mutation rates, and not structural variations in nucleic acid sequences on a large scale (Habig et al., 2021).

When microorganisms are first exposed to a stressful environment, it may enter a state of self-protection and exhibit slow or stagnant growth. Thus, microorganisms may regulate growth through epigenetic modifications. Among the known acetylated lysine residues, histone H4 carries four lysines in its N-terminal tail, namely lysines 5, 8, 12, and 16 (K5, K8, K12, and K16). Acetylation of the H4K16 site was found to regulate important processes such as gene silencing and transcription (Oppikofer et al., 2011). Methylation of specific histone-like residues can turn on or repress transcription in microorganisms (Lämke et al., 2016; Fabrizio et al., 2019). Subsequently, the microbe slowly undergoes acetylation or methylation modifications to affect gene expression until normal growth is restored.

Epigenetic modifications can adjust DNA structure, and alter the polyadenylation (pA) site where precursor mRNA is cleaved and polyadenylated, enabling cells to rapidly respond to environmental stress (Kaczmarek Michaels et al., 2020). Multiple pA sites are commonly found in eukaryotes, and these pA sites are present within cis-regulatory elements, introns and coding sequences. Different pA sites determine separate sites of polyadenylation, during which proteins with different levels of expression, structure, function and subcellular localization are produced, allowing the cell to respond better to different environmental stimuli (Tian and Manley, 2017). In other words, short-term adaptation may have altered the metabolic state of the microbe by regulating gene expression and maintaining this state within subsequent generations, a change similar to epigenetic regulation. Thus, when microorganisms undergo short-term adaptation, epigenetic modifications may reshape the structure of the genome, globally regulating the metabolic capacity of microorganisms and helping them to produce transient memories to cope with a change in environments.

### Cross-Protection Against Stress and Mechanisms

Microorganisms have complex and efficient regulatory networks that respond to changes in the culture environment. Microorganisms that have been adapted in a stressful environment are able to increase their stress tolerance. Interestingly, growth advantages can also be shown when switching to other stressful (Figure 2C). Moreover, the tolerance of microorganisms to multiple stresses is a ubiquitous phenomenon. In a high concentration of 500 mg/L corn steep liquor, *Lactobacillus rhamnosus* was adapted for 1 month to obtain an excellent strain. In addition to maintaining high antioxidant activity, antibacterial activity and antibiotic resistance similar to the original strain, it also showed relatively faster growth, greater resistance to acids, bile salts, lysozyme and 0.4% phenol (Wu et al., 2020). In the cobalt-containing stress environment, *S. cerevisiae* was adapted continuously or in stages. Compared with the wild-type strain, the adapted strain not only improved tolerance to cobalt, but also metals such as nickel, zinc and manganese, as well as high temperatures and hydrogen peroxide (Çakar et al., 2009). In prokaryotes, *Tetragenococcus halophilus* underwent short-term acclimation at 45°C for 1.5 h. Not only did the adaptability to high temperature improve, but its ability to resist osmotic stress also increased. When *T. halophilus* was transferred to a 10% ethanol environment and cultivated for 2.5 h, its survival rate increased by sevenfold (Yang et al., 2021). Similarly, short-term adaptation of the eukaryotic microorganism *Rhodotorula mucilaginosa* at 40°C for 30 min not only increased high temperature tolerance, but also enhanced tolerance to H_2_O_2_, NaCl and high heat stress, and the survival rate of their cells increased about 1.2-fold (Cheng et al., 2016). It was also found that *Candida glabrata*, an opportunistic fungal pathogen, was also adapted at high temperatures to enhance tolerance to other environmental stresses (Roetzer et al., 2011; Huang et al., 2019).

The mechanism in which microorganisms developed tolerance to other stresses after adaptation to one stress is unclear, and the enhancement of these tolerance traits may be affected by the interaction of genes and environments. The cause of microbial cross-tolerance may also be the common stimulation targets or the same ability to excrete harmful substances under different stress environments. *T. halophilus* strains subjected to high temperature stress not only improved their temperature tolerance but also ethanol tolerance (Yang et al., 2021). Ethanol is a toxicity factor that disrupts cell metabolism and damages the cell plasma membrane (Wijesundara and Rupasinghe, 2019). However, microorganisms can use membrane transport and take up extracellular organic compounds to improve stress tolerance under different stress conditions (Tang et al., 2014; Huang et al., 2016). Analysis revealed that the membrane protein insertion enzyme YidC of *T. halophilus* strains was significantly up-regulated after high temperature acclimation, which may be beneficial to the absorption of extracellular nutrients (Yang et al., 2021). Therefore, high temperature may enhance the performance of *T. halophilus* cytoplasmic membrane, thereby increasing resistance to ethanol.

After *T. halophilus* was acclimated to high temperature, the phosphate carrier protein HPr in the phosphotransferase system was significantly up-regulated. Previous studies showed that *T. halophilus* also induced the expression of *HPr* during acid acclimation, and then the strain was able to rapidly tolerate stress when encountering acid stress (He et al., 2016). Thus, the up-regulation of *HPr* expression may help microorganisms resist various stresses (He et al., 2016; Gao et al., 2019). Under ethanol stress, *HPr* up-regulation may also enhance the resistance of *T. halophilus* to acetic acid. In addition, the synthesis of ribosomal protein was accelerated when *Zygosaccharomyces rouxii* was acclimated to high temperatures, which not only enhanced its adaptability to high temperature, but also its ability to resist osmotic stress (Wang et al., 2021a). When yeast is acclimated to high temperature, it will induce the expression of a series of heat shock proteins Hsps (Kitichantaropas et al., 2016). The expression of Hsps proteins may be specific to various stresses, such as temperature, pH, ethanol stress, osmotic pressure, desiccation stress, antifungal and oxidative stress. Therefore, it is possible to simultaneously resist multiple environmental stresses through the regulation of yeast Hsps proteins.

After high-temperature adaptation of *C. glabrata*, the strains screened showed convergence in evolutionary phenotypes and the mutation in CgSTE11 was found to play a major role in high-temperature tolerance and in the observed cross-stress to other environmental stressors (Huang et al., 2019). The CgSTE11 mutation was also significantly more resistant to heat and acetic acid (Huang et al., 2019). It can be seen that mutations in genes affect the stress tolerance of microorganisms. The combination of different variants may also result in different phenotypes compared to single mutants. There are potentially millions of genes and gene combinations in eukaryotes. Gene-to-gene regulation alters the phenotype of microorganisms, while more appropriate getting gene coupling can be selected for stressful environmental conditions. Hence, the interaction between genes changes through genetic variation when the survival of microorganisms is under environmental stress, resulting in the mutual coordination between genes and the environment. The effects of gene-by-environment on yeast were studied by alternative carbon sources, osmotic stress and genotoxic stress in 14 different environments and 4,000 yeast single mutants (Costanzo et al., 2021). The relationships that regulate the global network of genetic interactions in yeast under different environmental stresses were revealed, and some genes with close functional links were also identified, which have implications for enhancing tolerance to different environmental stresses.

The same stress resistance mechanism may also exist between different microorganisms. The PhoP/PhoQ two-component system is a regulatory system shared among a variety of microorganisms (Wang et al., 2019; Wei et al., 2019; Yeom and Groisman, 2021). This system controls the resistance of several Gram-negative bacteria to toxic substances, Mg^2+^, acidic environments, and cationic antimicrobial peptides (Groisman Eduardo et al., 2021). The PhoP/PhoQ two-component system consists of the DNA-binding protein PhoP and the sensor PhoQ (Yeo et al., 2012). PhoQ protein can change the phosphorylation state of PhoP, respond to signal changes outside the cytoplasm, and change the resistance to the external environment (Sanowar and Le Moual, 2005). In short, microorganisms may enhance tolerance in different stressful environments through the same regulatory approach. Therefore, an in-depth study of cross-tolerance mechanisms is important to improve microorganisms’ ability to resist environmental stresses.

## Conclusion and Future Perspectives

We found that long- or short-term adaptation of microorganisms can improve the utilization of non-preferred substrates and adaptability of stress environments. Therefore, adaptation is universal to microorganisms. A number of mechanisms may cause increased stress tolerance in microorganisms, including the presence of degradable proteins in microorganisms that improve environmental adaptation, epigenetic modifications on histones of microorganisms, and cross-tolerance, allowing microorganisms to simultaneously respond to different stressful environments. Thus, the adaptation mechanisms of microorganisms are diverse. The analysis of mechanisms is still unclear in existing studies, and further research is needed. With the development of high-throughput screening methods and bioinformatics technology, various epigenetic modifications are studied by proteomics. In addition, transcriptomics was used to study the cooperative regulation between genes while the effects of LncRNAs and MicoRNAs on epigenetic modifications were investigated. The use of these tools for in-depth research into the mechanisms of adaptation expands the uncharted territory of biology, and facilitates the rational design of microorganisms in industrial production.

## Author Contributions

Y-ST conceived and drafted the manuscript. B-ZL, Z-HL, and R-KZ revised the manuscript. Y-JY supervised the project. All authors read and approved the final manuscript.

## Conflict of Interest

The authors declare that the research was conducted in the absence of any commercial or financial relationships that could be construed as a potential conflict of interest.

## Publisher’s Note

All claims expressed in this article are solely those of the authors and do not necessarily represent those of their affiliated organizations, or those of the publisher, the editors and the reviewers. Any product that may be evaluated in this article, or claim that may be made by its manufacturer, is not guaranteed or endorsed by the publisher.
